# Role of critical elements in botulinum neurotoxin complex in toxin routing across intestinal and bronchial barriers

**DOI:** 10.1371/journal.pone.0199524

**Published:** 2018-07-05

**Authors:** Koyel J. Ghosal, Kruti Patel, Bal Ram Singh, Martha L. Hale

**Affiliations:** 1 Department of Chemistry and Biochemistry, University of Massachusetts Dartmouth, North Dartmouth, Massachusetts, United States of America; 2 Molecular and Translational Sciences Division, United States Army Medical Research Institute of Infectious Diseases, Fort Detrick, Maryland, United States of America; Institut Pasteur, FRANCE

## Abstract

The highly potent botulinum neurotoxin serotype A (BoNT/A) inhibits neurotransmitter release at neuromuscular junctions resulting in flaccid muscle paralysis, respiratory arrest and death. In order to reach their neuronal cell targets, BoNT/A must cross epithelial cell barriers lining the intestines and airways. The toxin is produced as a large protein complex comprised of the neurotoxin and non-toxic neurotoxin-associated proteins (NAPs). Although NAPs are known to protect the toxin from harsh environments, their role in the movement of BoNT/A across epithelial barriers has not been fully characterized. In the current study, movement of the toxin across epithelial cells was examined macroscopically using a sensitive near infrared fluorescence transcytosis assay and microscopically using fluorescently labeled toxin and confocal microscopy. The studies show that the BoNT/A complex internalizes more rapidly than the pure toxin. The studies also show that one NAP protein, hemaglutinin 33 (Hn33), enhanced both the binding and movement of a deactivated recombinant botulinum neurotoxin A (DrBoNT) across epithelial cell monolayers and that the toxin associates with Hn33 on the cell surface. Collectively, the data demonstrate that, in addition to their protective role, NAPs and Hn33 play an important role in BoNT/A intoxication.

## Introduction

Botulinum neurotoxins (BoNTs), produced primarily by the anaerobic bacterium, *Clostridium botulinum*, cause the often fatal disease botulism [1]. BoNTs are highly potent toxins, with a calculated LD_50_ in humans estimated to be 1 μg/kg body weight when administered orally, 1.3–2 ng/kg body weight when given intravenously and 10–13 ng/kg body weight when introduced via aerosol [1, 2]. There are seven serotypes, of which the prototype BoNT/A is the most relevant toxin causing human disease [3]. As with other serotypes, BoNT/A is a two-chain polypeptide composed of a 100 kDa heavy chain (Hc) and a 50 kDa light chain (Lc). The Hc binds the SV2 protein located on the peripheral neuronal presynaptic membranes and initiates internalization of the Lc protease.

The substrate of Lc, the 25 kDa synaptosomal-associated protein (SNAP-25), is located at the presynaptic membrane of neuromuscular junctions. Cleavage of SNAP-25 prevents the presynaptic vesicles from attaching to the cell membrane, thereby inhibiting the release of acetylcholine at neuromuscular junctions. In the absence of neurotransmitter release, muscles are not innervated and become unresponsive, resulting in paralysis. In the case of botulism, the muscles of the diaphragm are paralyzed resulting in respiratory arrest and death.

BoNTs are released from the bacterium in the form of protein aggregates, referred to as progenitor toxin complexes (PTCs), that are comprised of the toxin non-covalently bound to non-toxic proteins [4]. These neurotoxin-associated proteins (NAPs), including both non-hemagglutinin proteins and proteins possessing hemagglutinin properties, protect BoNTs from proteolytic digestion in the gastrointestinal tract as well as from other adverse environmental conditions that the toxin might encounter [5, 6]. Whether due to enhanced stability or active participation in the intoxication process, the PTCs are more potent, both *in vitro* and *in vivo*, than the purified toxin alone [7, 8].

Other than wound botulism, in which the toxin is essentially injected into the bloodstream, there are two stages required for intoxication. BoNTs are initially absorbed at the apical surface of epithelial cells lining the intestines or airways, taken across the epithelial cell barrier, and released at the basolateral surface. BoNTs are then distributed through the general circulatory system from which they reach peripheral nerves enter neuronal cells by receptor-mediated endocytosis [9–13]. While there is little doubt that BoNTs cross epithelial cell barriers, the importance of NAPs in toxin transport remains a point of contention. Investigations show that the purified toxin crosses both intestinal and pulmonary epithelial cell barriers by transcytosis while other investigators suggest that NAPs adsorb onto epithelial cells and facilitate BoNT passage by a paracellular route [10, 11, 14, 15].

Although BoNTs may utilize either a paracellular route or transcytosis as a means to enter the body, the fact that most intoxications occur by PTC ingestion, with the onset of symptoms reported to occur as early as 2 h [2] after ingestion, suggests that NAPs associated with BoNT may contribute significantly to the toxin moving across the epithelial cell barriers [16–18]. Therefore, characterization of NAPs role in BoNT transcytosis becomes important to understand early events in BoNT intoxication.

BoNTs are classified as a Category A biothreat agent (Centers for Disease Control, Atlanta, GA) because PTCs are easy to produce, highly potent, and easily dispersed; in addition, there is no vaccine or therapeutic agent currently available. While not infectious, the stability of BoNT PTCs makes them easily aerosolized for inhalation delivery and a serious threat as a bioterror agent [2]. Thus, determining whether NAPs influence BoNT crossing pulmonary epithelial cell barriers becomes critical for understanding and developing novel therapeutics against the intoxication process. In order to determine whether NAPs influence BoNT entry across epithelial cells, experiments were performed using a catalytically-deactivated recombinant botulinum neurotoxin A (DrBoNT) which was developed by mutating two glutamic acid residues at the active site, that rendered the molecule non-toxic [13, 19, 20]. The nontoxic DrBoNT has been shown to be identical to BoNT/A in structure and antigenic properties. Therefore, DrBoNT was chosen for these studies, in order to minimize exposure to the toxic BoNT/A. DrBoNT was used to compare BoNT transcytosis either alone, in presence of NAPs (BoNT/A complex) or in the presence of hemagglutinin 33 (Hn33). Hn33, a 33 kDa protein, is the most abundant NAP component in the BoNT/A complex and is known to protect the toxin against proteases [21], as well as facilitate BoNT translocation across epithelial barriers [8, 15]. Using a sensitive near infrared (NIR) fluorescence transcytosis assay [22] and laser scanning confocal microscopy, Hn33 was shown to facilitate transcytosis of DrBoNT across polarized human bronchial epithelial (HBE) cell monolayers in a manner similar to BoNT/A complex crossing intestinal epithelial cells. The results presented here show that BoNT/A crosses polarized epithelial cell monolayers by receptor-mediated transcytosis, but that the addition of Hn33 facilitates BoNT/A movement across the cell, possibly by allowing the toxin to bind to Hn33 and move across the cell by adsorptive transcytosis.

## Materials and methods

### Cells and reagents

Human bronchial epithelial cells (16HBE14o-) were obtained from Dr. D. C. Gruenert, California Pacific Medical Center Research Institute, San Francisco, CA [23]. Human intestinal epithelial cells (HT-29), McCoy’s 5A medium, Eagle’s minimal essential medium (EMEM) and Hank’s balanced salt solution (HBSS) were purchased from American Type Culture Collection (Manassas, VA). Bronchial epithelial growth medium (BEGM), Reagent Pack subculture kit containing HEPES buffer, trypsin, and trypsin neutralizing solution were purchased from Lonza Walkersville (Walkersville, MD) while FBS was purchased from Hyclone Laboratories, Logan, UT, and heat-inactivated (56°C for 30 min) before use. Penicillin/streptomycin solution, L-glutamine, AlexaFluor 680, AlexaFluor 488, Pacific Blue, Texas Red Phalloidin, Hoeschst dye 33342, plasma membrane and nuclear labeling kits, supplies for SDS-PAGE, micro-BCA assays, and 10,000 MW dextran labelled with Alexa 680 were purchased from Invitrogen (Carlsbad, CA). The 800 CW antibody-labeling kit was purchased from LI-COR Biosciences (Lincoln, NE); Endohm chambers and an EVOM resistance meter were obtained from World Precision Instruments (Sarasota, FL).

### Expression and purification of BoNT/A, BoNT/A complex, DrBoNT and Hn33

BoNT/A and BoNT/A complex were purified from *C*. *botulinum* (strain Hall) cultures as previously described [24]. DrBoNT and recombinant Hn33 were expressed and purified as described previously [19, 25].

### Labeling proteins

For transcytosis experiments, DrBoNT was labeled with the NIR 800 CW dye (DrBoNT-800) while recombinant Hn33 was labeled with AlexaFluor 680 dye (Hn33-680). For confocal imaging analysis, BoNT/A, BoNT/A complex, or DrBoNT were labeled with AlexaFluor 488 (BoNT/A-488, BoNT/A complex-488, DrBoNT-488); and Hn33 with Pacific Blue (Hn33-PacBlue). All labeling followed the protocols provided by the vendors. Labeled proteins were dialyzed to remove free dye from the solution and protein concentrations of labeled proteins were compared to that of unlabeled proteins using a micro-BCA assay and densitometry of protein after SDS-PAGE and Coomassie blue staining. When compared to the unlabeled protein, concentrations of the labeled protein were similar.

Proteins labeled with fluorescent dyes have been previously used to study transcytosis [26–28] and have shown proteins labeled with fluorescent dyes provide a sensitive method for measuring transcytosis. Experiments performed in this study used proteins that were conjugated to dyes that fluoresce in the NIR range and were measured using the Odyssey imaging system as previously described [22, 29]. The NIR dyes emit at 700 nm and 800 nm and significantly reduces auto-fluorescence common with dyes that fluoresce in the 400 nm to 600 nm range. The imaging system [30, 31] (https://www.licor.com/bio/products/imaging_systems/odyssey) has a wide dynamic range and with a high signal-to-noise ratio which improves the sensitivity of the fluorescence assay.

### Transcytosis of DrBoNT and Hn33 through polarized HBE cell monolayers

HBE cells were grown to 80% confluence and sub-cultured in EMEM supplemented with 10% FBS. All experiments in this study were performed on HBE cells between 4–15 passage levels. For transcytosis experiments, cells were trypinsized using the Reagent Pack subculture system, resuspended (5 x 10^5^ cells/ml) in BEGM and 500 μl added to collagen-coated inserts (1.0 mm, 24 well plates, BD Biosciences, Farmington MA). Medium in both the apical and basal chambers was changed every other day and transepithelial electrical resistance (TEER) of the cell monolayer was measured using an endohm chamber. By day 7, the TEER had reached 800–1400 ohms/cm^2^ range. On day 7, apical medium was replaced with media containing 0.25 μg/ml 10,000 MW Dextran-680 Alexa dye (DEX-680). Inserts were incubated (37°C, 5% CO_2_) for an additional 18–24 h, and then transferred to another 24-well plate containing 800 μl/well of BEGM medium. The original plate was scanned on the Odyssey Imaging System in order to detect leakage of the dextran into the basal chamber [32]. Only inserts with TEER measurements of 800–1200 ohm/cm^2^ and that did not show leakage of the DEX-680 from the apical to the basal side, were used for transcytosis experiments.

Transcytosis experiments were performed by replacing the media in the apical chamber with 500 μl BEGM containing varying concentrations of the labeled proteins, followed by incubation at 37°C, 5% CO_2_. At designated times, 60 μl aliquots were removed from the basal medium, placed in a black microtiter plate with clear bottoms, and scanned on the Odyssey Imaging System. Concentrations of DrBoNT and Hn33 in the basal medium were calculated using a DrBoNT-800 or Hn33-680 standard curve and ρ values determined by an unpaired Student’s t-test. Results were considered significant at ρ <0.05.

### Sample preparation for confocal microscopy

#### a) Determination of tight junction integrity

Polarized monolayers of HBE cells, cultured on 0.02 μm Anopore cell culture inserts (Sigma-Aldrich, St. Louis, MO), were examined by confocal microscopy to determine whether BoNT/A or Hn33 altered the integrity of tight junctions. DrBoNT-800 (150 nM) and Hn33 (666.7 nM) were added to the apical side of inserts and incubated for 24 hrs (37°C, 5% CO_2_). Cells were rinsed with PBS, pH 7.4, fixed in pre-warmed (37°C) PBS containing 3.7% paraformaldehyde, and then washed with PBS. Permeabilization of the plasma membrane was performed by soaking the inserts (15 min, 25°C) in 0.2% Triton X 100 (Sigma Aldrich, St. Louis, MO). In order to block non-specific binding sites, 2% IgG-Free Protease Free BSA prepared in PBS (2% BSA-PBS) was applied to the inserts (30 min, 37°C). The primary antibody, ZO-1, was diluted in 1% BSA-PBS and then layered onto the cell monolayer. After 1 h incubation (37°C), monolayers were washed 3 times in PBS. The secondary antibody AlexaFluor 488 goat anti-rabbit IgG, diluted in 1% BSA-PBS, was added to the cells and incubated for 1 h at 37°C. Texas Red Phalloidin was added for visualization of the actin filaments. The inserts were washed in PBS followed by the addition of Hoechst dye 33342 for visualization of nuclei. The inserts were then washed twice in PBS. To view the cells using confocal microscopy, membranes were removed from the insert, mounted onto microscope slides (Daigger, Vernon Hill, IL) using Fluoromount G mounting medium (Fisher Scientific, Suwanee, GA) and then covered with a cover glass (Fisher Scientific, Pittsburgh, PA). The cells were then imaged on a BioRad Radiance 2000 MP laser scanning confocal system (Hemel Hempstead, UK) connected to a Nikon (Melville, NY) TE 300 inverted microscope and green and red fluorescence was visualized using 488 nm and 568 nm wavelength excitation, respectively, from a krypton/argon laser while nuclei were imaged using a two-photon excitation from a Ti/sapphire laser tuned to 780 nm. Images were saved using BioRad Laser Sharp software.

#### b) Tracking DrBoNT and Hn33 binding and internalization in HBE polarized cells

HBE cell monolayers were cultured on Anopore cell culture inserts for 5–7 days to ensure polarization of the cell monolayer. DrBoNT-488 (150 nM), Hn33-PacBlue (700 nM) or both proteins were added to 500 μL BEGM media in the apical chamber. After incubating (37°C, 5% CO_2_, 95% humidity) the cells for various times, media was discarded from the inserts and cells were rinsed and fixed as described in the previous section. Plasma membrane labeling was performed by incubating the cell monolayers (10 min, 37°C) with wheat germ agglutinin-594 (WGA-594) solutions (5 μg/ml in HBSS). Membranes were removed from the inserts and mounted onto microscope slides using VectaShield mounting medium (Vector Laboratories, Inc., Burlingame, CA).

#### c) Tracking binding and internalization of BoNT/A and BoNT/A complex in HT-29 cells

HT-29 cells were maintained in McCoy’s 5A media supplemented with 10% FBS. For confocal microscopy experiments, cells were seeded onto cover glasses (Fisher Scientific, Pittsburg, PA) and grown (37°C, 5% CO_2_) to confluency. Cells were treated with 300 nM BoNT/A-488 or BoNT/A complex-488. After incubation (37°C, 5% CO_2_) for various times, the cells were washed twice with PBS, pH 7.4, pre-warmed to 37°C and fixed (15 min) with freshly prepared PBS containing 3.7% paraformaldehyde. Cells were washed twice with HBSS and incubated (10 min) with WGA-594 to label the plasma membrane. Cells were then washed twice with PBS and mounted onto a glass slide using 25 μl of mounting media (Biomeda Corp., Foster City, CA).

### Imaging cells using confocal laser scanning microscopy

Labeled cells were imaged using a Zeiss LSM 710 confocal laser scanning microscope (CLSM) (Zeiss, Thornwood, NY). Blue (Ex/Em– 410/455), green (494/519) and red (590/617) fluorescence was visualized using a 405 diode laser, an argon laser, and a HeNe laser, respectively. A 20X objective (Zeiss, Plan-Apochromat 20X/0.8) was used for routine imaging and a 63X objective (Zeiss, Plan-Apochromat 63X/1.4 Oil, DIC) when performing optical sectioning of cells along the Z-axis, which was accomplished by scanning the cells using appropriate lasers on an XY-plane at each interval of approximately 0.3 μm optical thickness along the Z-axis. The stack of all the optical slices thus obtained along the Z-axis in a selected scan area collectively formed the Z-stack, with which a three dimensional image of the cells was digitally reconstructed. Each optical slice from the Z-stack provides information from the respective Z-section of the cells, thus enabling the visualization of all labeled proteins and cell components within that Z-section. The pixel format used was 512 X 512. The Zeiss Zen 2008 software was used for imaging and all image analysis was carried out using the Zeiss Zen 2011 software and ImageJ software (https://imagej.nih.gov/ij/). Colocalization studies were carried out using the Fiji(ImageJ) software (https://fiji.sc/) and Manders coefficient was used to determine the percentage overlap [33].

### Binding affinity of Hn33 to DrBoNT

Binding of Hn33 to DrBoNT was assessed using surface plasmon resonance (SPR). SPR data was collected using Biacore T100 instrument (GE Healthcare Bio-Sciences, Pittsburgh, PA). A CM_3_ sensor chip was primed with PBS pH 7.4 followed by 50 mM sodium hydroxide solution prior to immobilization of Hn33 onto the chip surface. The chip was subsequently activated with 0.2 M (1-Ethyl-3-(3-dimethylaminopropyl)-carbodiimide) (EDC), 0.05 M N- hydroxysuccinimide (NHS) solution. The 300 nM ligand Hn33 was immobilized on the surface using the amines coupling by injections for 7 min at 10 μl/min flowrate. After ligand coupling, the chip was deactivated with 1 M ethanolamine (pH 8.5) and primed twice with binding buffer PBS. Different concentrations of 450, 300, 200, 133, 89, 59, and 30 nM of analyte DrBoNT were injected over the sensor surface with 5 min association time and 10 min dissociation time. The raw data was processed and analyzed to determine the binding constant using BIA Evaluation software, and report tables were analyzed with Microsoft excel.

## Results

### DrBoNT and Hn33 did not affect tight junction integrity

**Fig 1:**
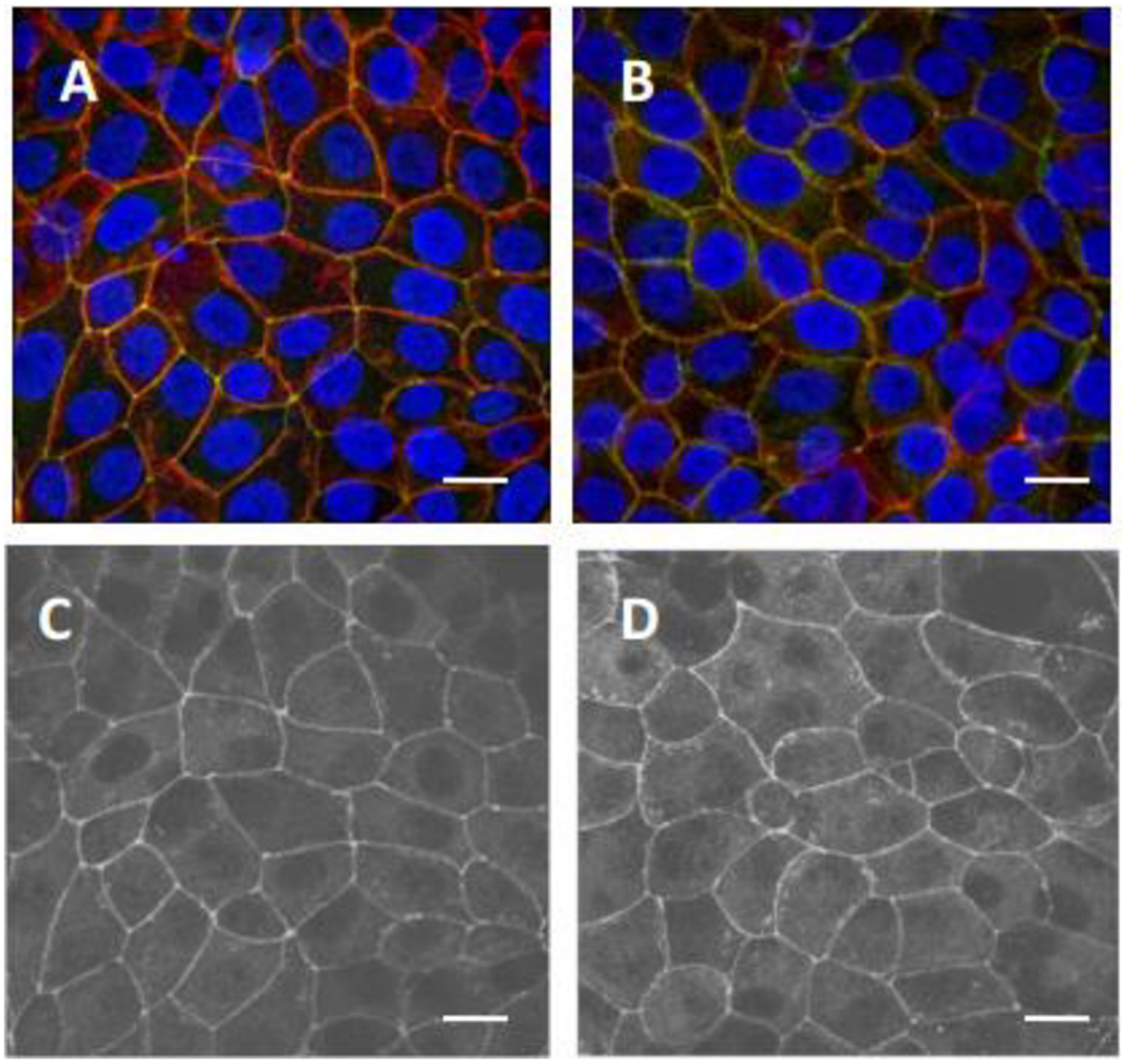
Confocal microscopic images showing effects of DrBoNT and Hn33 on HBE cell monolayer integrity. HBE cells were cultured on transwell inserts until polarized monolayers developed. DrBoNT-800 (100 nM) and Hn33-680 (600 nM) were added to the apical medium and incubated for 24 h. Cell monolayers were stained with an antibody that recognizes the tight junction protein ZO-1 (green). Nuclear DNA was stained with Hoechst 33342 (blue) and actin with Texas Red phalloidin (red). Cells were imaged using confocal microscopy. (A) Cell monolayers without DrBoNT and Hn33 (control); (B) cell monolayers treated with DrBoNT and Hn33; C and D: Gray-scale images of A and B respectively, showing tight junctions. White bar = 10 μM.

Integrity of the cell monolayer was assessed using 4 different criteria: TEER measurements, leakage of DEX-680 from the apical into the basal medium [23], monitoring the level of the apical medium [34], and microscopic examination of the cell monolayers. Addition of the proteins to the apical medium did not decrease TEER measurements nor increase leakage of dextran from apical to basal medium at any time during 1–24 h (data not shown). Additionally, when examined microscopically, cell monolayers were intact with no visible loss of monolayer integrity during the 24 h experimental period. Further examination of cell monolayer tight junctions using confocal microscopy showed no loss of membrane integrity 24 h after treatment with DrBoNT and Hn33 (Fig 1). As shown in the photograph of the confocal image, the tight junction protein, ZO-1 (green fluorescence), was visualized around the periphery of cells in both control and treated cell monolayers, indicating that tight junctions of the cell monolayer had not been compromised. There were no differences observed in the morphology of both nuclei (blue fluorescence) and actin (red fluorescence) between the control and treated cell monolayers, further indicating that the treatments did not affect cell morphology.

### DrBoNT and Hn33 crossed HBE cell monolayers by transcytosis

**Fig 2:**
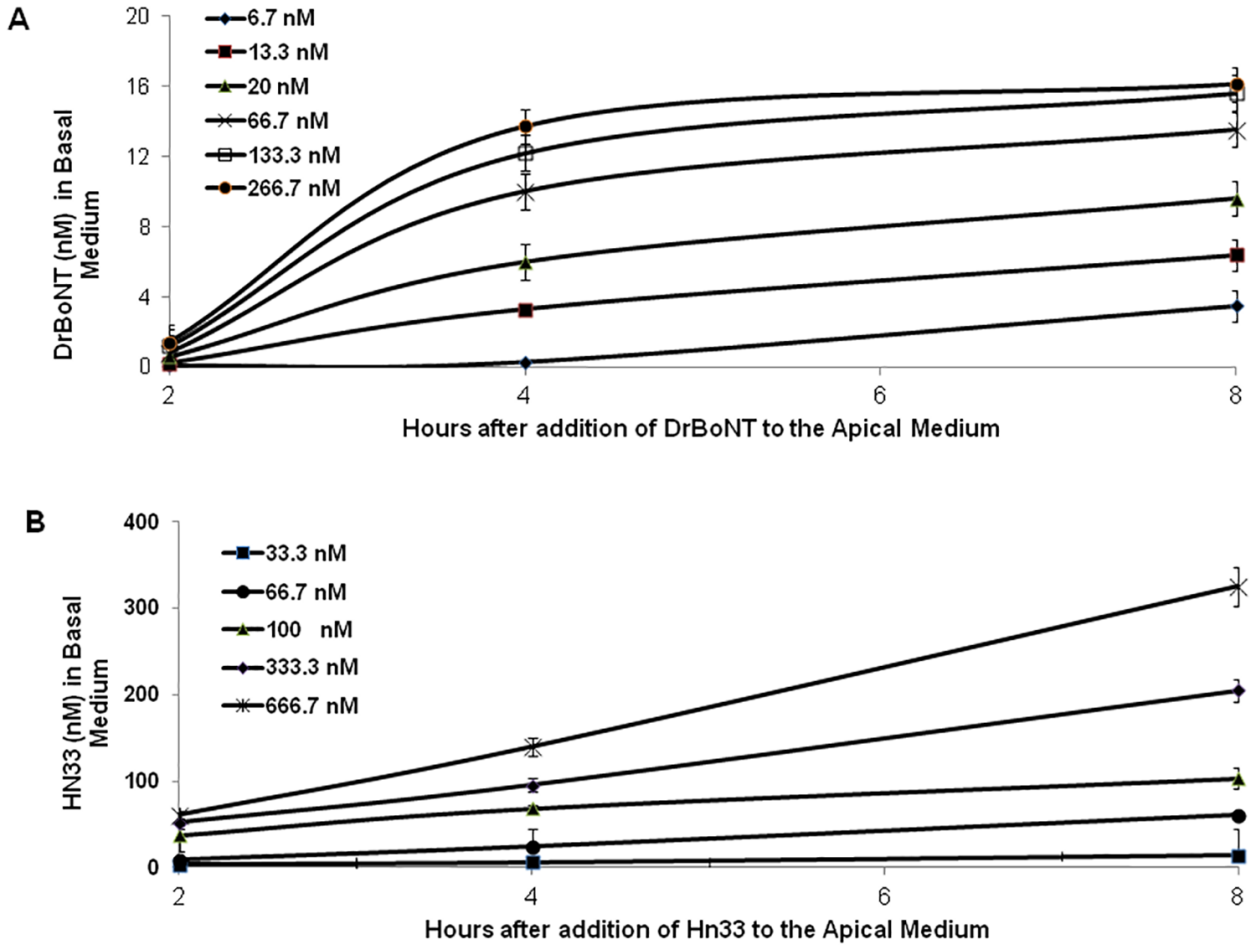
Movement of DrBoNT and Hn33 across polarized HBE cell monolayers via transcytosis. Varying concentrations (A) DrBoNT-800 (6.6–266.7 nM) or (B) Hn33-680 (33–666.7 nM) were added to the apical medium on HBE cell monolayers. After 2, 4, and 8 h incubations (37°C, 5% CO_2_), aliquots of the basal medium were scanned on an Odyssey imaging system. Labeled protein standards were used to convert density measurements to nM concentrations. Results were the average of three inserts ± standard deviation. Statistical analysis was performed using a Student’s t-test.

Similar to previous investigations, in which BoNT/A was shown to cross epithelial cells via transcytosis [35–37], the investigations presented here showed that DrBoNT crossed HBE cell monolayers via transcytosis (Fig 2A). The amount of DrBoNT detected in the basal medium showed that the rate of transcytosis depended upon the initial DrBoNT concentration added to the apical medium although the amount of DrBoNT levels off at higher concentrations (Fig 2A, 133.3 and 266.7 nM), suggesting that BoNT/A trancystosis may occur by receptor-mediated mechanisms [14]. DrBoNT detected in the basal medium of the 133.3 and 266.7 nM concentrations were not statistically significant from each other (ρ > 0.05); DrBoNT detected in the basal medium of the remaining concentrations was statistically significant (ρ < 0.05). While the amount of Hn33 detected in the basal medium was also dependent upon the initial Hn33 concentration added to the apical medium (Fig 2B), Hn33 movement across cell monolayers did not level off at higher concentrations (Fig 2B, 333.3 and 666.7 nM) and differences between each concentration were statistically significant (ρ <0.05). These results suggest that Hn33 does not require a membrane receptor, but relies upon adsorption to the cell surface [38, 39].

### Hn33 facilitated DrBoNT transcytosis across HBE cells

**Fig 3:**
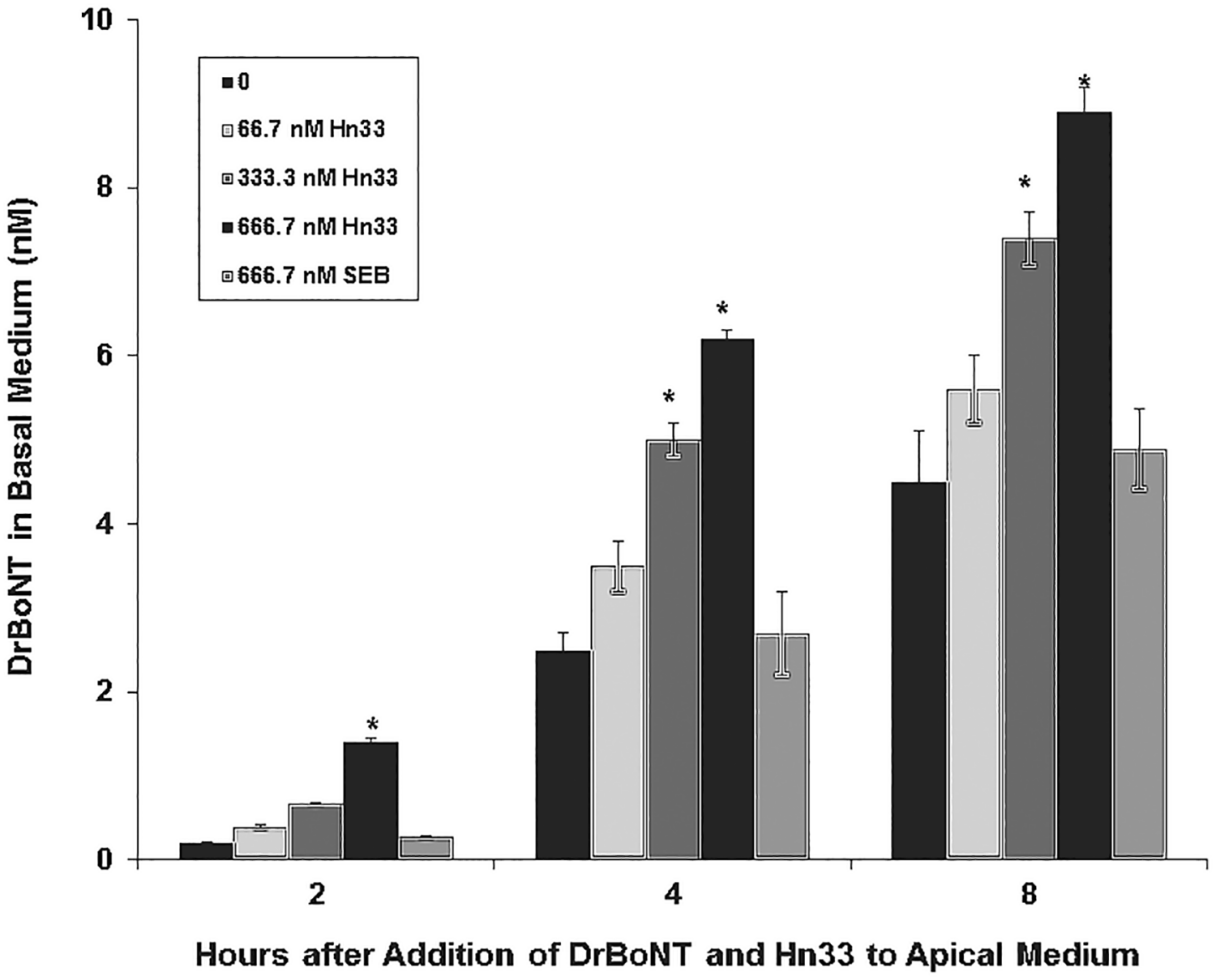
Effect of Hn33 on DrBoNT transcytosis across HBE cell monolayers. A constant amount of DrBoNT-800 (13.3 nM) alone, or varying concentrations of Hn33 (66.7–666.7 nM) were mixed before adding to the apical medium. SEB was added instead of Hn33 as a negative control. After incubating at 37°C, 5% CO_2_, aliquots of basal medium were removed and scanned on the Odyssey imaging system. Results were the average of three inserts ± standard deviation. Significant differences (ρ<0.05) between BoNT alone and BoNT with Hn33 are noted (*).

A constant amount of DrBoNT was mixed with varying concentrations of Hn33-680 and added to the apical side of polarized HBE cell monolayers. At designated time points, aliquots of the basal medium were removed and scanned on the Odyssey imaging system. The amount of DrBoNT detected in the basal medium increased in proportion to the amount of Hn33 added (Fig 3). When Hn33 (666.7 nM) was mixed with DrBoNT (13.3 nM), the amount of DrBoNT detected in the basal medium at each time point tested (2–8 h) was 3–5 times greater than that detected with DrBoNT alone. The facilitation effect that Hn33 exerted upon DrBoNT transcytosis was specific since Staphylococcal enterotoxin B (SEB) with a similar molecular weight (30 kDa) as Hn33 (33 kDa) did not show the same effect.

When DrBoNT transcytosis was compared to that of DrBoNT mixed with the highest Hn33 concentration (666.7 nM), the increase of DrBoNT transcytosis was substantially different (over 6-fold; ρ < 0.05) even at 2 h, and was significantly different with the addition of the two higher Hn33 concentrations (333.3 nM and 666.7 nM) at 4 and 8 h (about 2-fold; Fig 3). DrBoNT did not have the same effect upon Hn33 because the addition of higher concentrations of DrBoNT to a constant Hn33 concentration did not affect Hn33 transcytosis (Table 1).

**Table 1:** Detection of Hn33-680 in basal medium after adding a constant amount of Hn33-680 and varying amounts of DrBoNT to the apical medium.

Amount (nM) added to the apical medium^a^		Time (h) after adding DrBoNT and Hn33 to the apical medium		
Hn33-680	DrBoNT	2	4	8
15	0	0.6±0.02^b^	1.2±0.4	3.4±0.6
15	3.3	0.7±0.2	1.3±0.5	2.9±0.7
15	133.3	0.7±0.1	1.2±0.4	2.8±0.5
15	266.7	0.6±0.04	1.1±0.3	3.4±0.5

^a^Varying amounts of DrBoNT were mixed with a constant amount of Hn33-680 before adding to the apical medium.

^b^Concentration (nM) of Hn33-680 detected in the basal medium. Results were the average of three inserts ± standard deviation. When compared to Hn33 alone, groups were not significantly different at ρ <0.05.

### DrBoNT underwent receptor-mediated transcytosis while Hn33 was adsorbed onto the cell membrane

To determine whether a specific receptor was involved in the attachment and movement of BoNT across HBE cells, a fixed amount of DrBoNT-800 was mixed with varying concentrations of unlabeled DrBoNT and added to the apical side of polarized HBE cells. The labeled protein was then detected in the basal medium using the Odyssey imaging system. When compared to labeled protein alone, the addition of unlabeled DrBoNT at the higher concentrations (133.3 nM or 266.7 nM) decreased the amount of DrBoNT-800 detected in the basal medium as analyzed by a one-way Student’s t-test at a ρ<0.05 (Table 2). The results of these experiments indicated that BoNT/A initiates transcytosis by binding to a receptor on the surface of HBE cells.

**Table 2:** Detection of DrBoNT-800 in basal medium after DrBoNT and unlabeled DrBoNT were added to the apical medium.

Amount (nM) added to the apical medium^a^		Time (h) after adding DrBoNT-800 and DrBoNT to the apical medium		
DrBoNT-800	DrBoNT	2	6	8
**3.3**	**0**	**0.1±0.0**^b^	**1.2±0.02**	**2.6±0.2**
**3.3**	**3.3**	**0.06±0.0**	**1.1±0.02**	**2.3±0.03**
**3.3**	**133.3**	**0.05±0.0**	*1.0±0.01	*1.7±0.03
**3.3**	**266.7**	*0.01±0.0	*0.9±0.04	*1.1±0.2

^a^Varying amounts of unlabeled DrBoNT were mixed with a constant amount of DrBoNT-800 before adding to the apical medium.

^b^Concentration (nM) of DrBoNT-800 detected in the basal medium. Results were the average of three inserts ± standard deviation.

* Significantly different (ρ<0.05)

Additionally, receptor mediated endocytosis requires energy, thus occurring at physiological temperatures [35]. When inserts with DrBoNT-800 in the apical medium were held at 4°C, transcytosis of DrBoNT was inhibited (data not shown) during the first 4 h (fluorescence detected in DrBoNT-800 inserts was ≤ background fluorescence). Further assessment was not performed because by 6–8 h, DEX-680 was detected in the basal medium indicating disruption of HBE cell monolayer integrity and thus transcytosis could no longer be tracked. The results are in agreement with the findings of other investigators [12, 14] who show that BoNT/A initiates transcytosis across HBE cell monolayers by binding to a specific receptor on the cell surface.

Unlike DrBoNT, Hn33 was not dependent upon binding to a specific cell receptor on the cell for transcytosis to occur. When varying concentrations of unlabeled Hn33 were mixed with a fixed amount of Hn33-680, the amount of Hn33-680 detected in the basal medium showed a very slight decrease with the addition of more unlabeled protein (Table 3) that were not statistically significant (ρ<0.05). These studies suggested that Hn33 does not require a specific membrane receptor for transcytosis.

**Table 3:** Detection of Hn33-680 in basal medium after Hn33-680 and unlabeled Hn33 were added to the apical medium.

Amount (nM) added to the apical medium^a^		Time (h) after adding Hn33 to the apical medium		
Hn33-680	Hn33	2	4	8
15	0	0.80±0.03^b^	1.8±0.04	2.4±.0.06
15	15	0.67±0.04	1.7±0.04	2.7±0.07
15	400	0.77±0.03	1.4±0.03	2.5±0.09
15	900	0.78±0.04	1.3±0.05	2.2±0.07

^a^Varying amounts of unlabeled Hn33 were mixed with a constant amount of Hn33-680 before adding to the apical medium.

^b^Concentration (nM) of Hn33-680 detected in the basal medium. Results were the average of three inserts ± standard deviation. Groups were not significantly different at ρ<0.05

Transcytosis studies (Fig 3) showed that Hn33 increases DrBoNT movement across polarized HBE cells, but does not examine the movement of DrBoNT across individual cells. Further studies were undertaken to assess the ability of Hn33 to facilitate DrBoNT binding and internalization in individual cells using confocal microscopy.

#### DrBoNT binding in the presence of Hn33 appeared similar to binding of Hn33 alone

**Fig 4:**
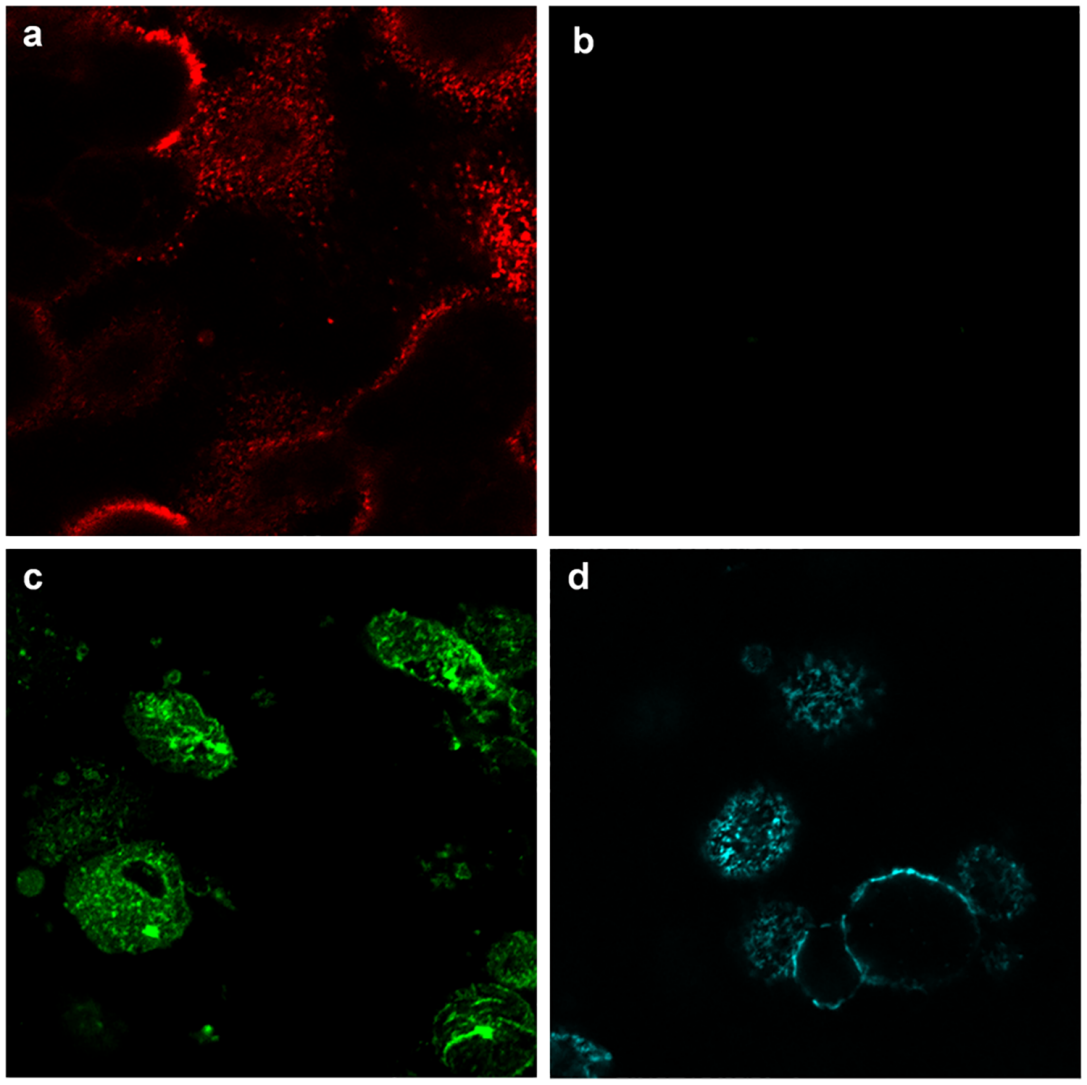
Binding patterns of DrBoNT and Hn33 at the cell surface. Polarized HBE cell monolayers were incubated with DrBoNT-488 (150 nM) in the absence or presence of Hn33 (700 nM) for 5 min, fixed and then imaged using confocal microscope. (a) WGA-594 binding to the cell surface; (b) DrBoNT-488 in the absence of Hn33; (c) DrBoNT-488 in the presence of Hn33; (d) Hn33-PacBlue alone. Images were obtained using a 63X oil immersion objective with a 2X magnification.

**Fig 5:**
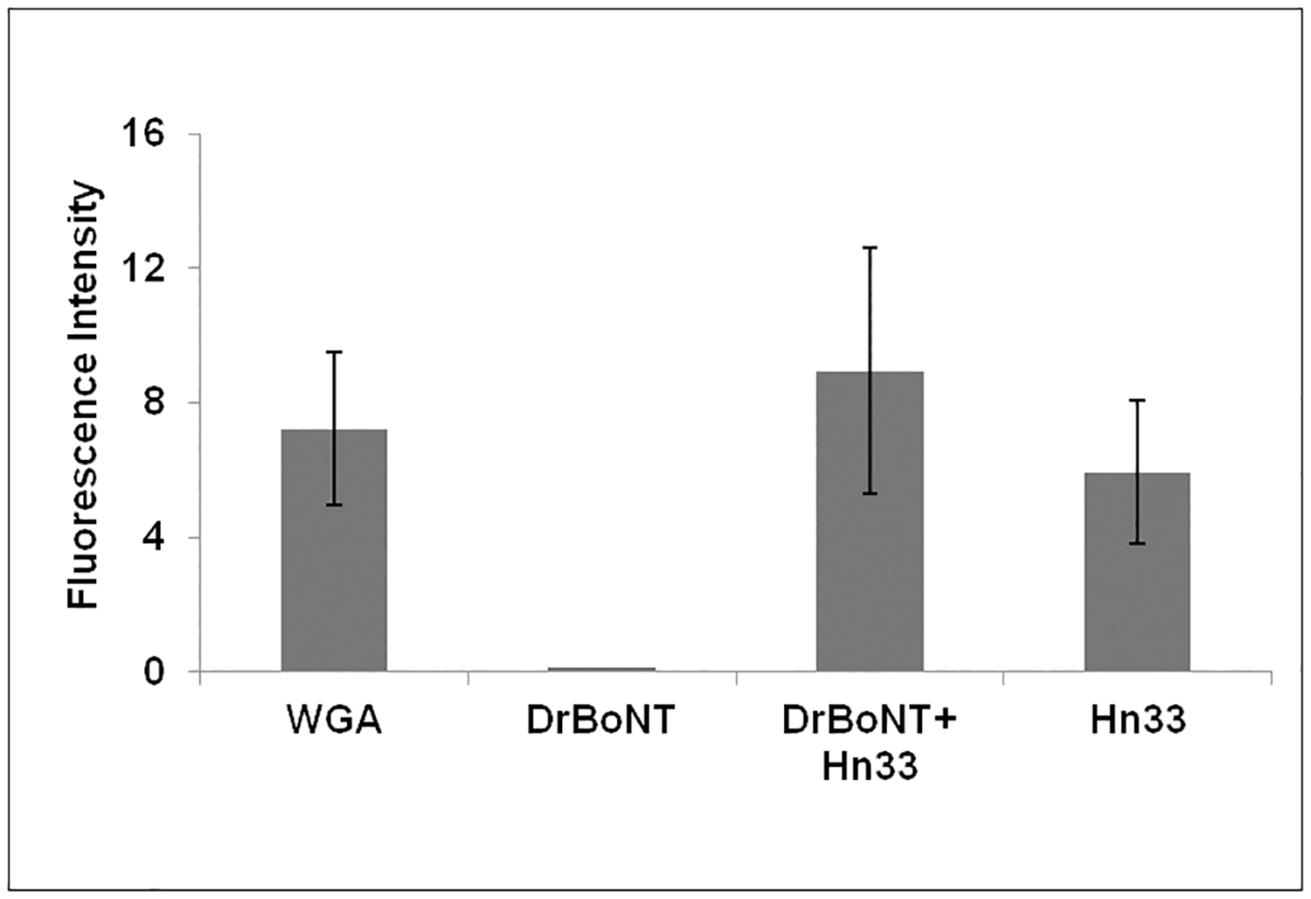
Comparison of cell binding of WGA-594, DrBoNT-488, DrBoNT with Hn33 and Hn33 alone. The mean fluorescence intensities of cells for each treatment were measured and the background subtracted. The results were the average of five cells ± standard deviation. Statistical analysis using an unpaired, two-tailed Student’s t-test showed that binding of DrBoNT alone was significantly different from that of WGA, DrBoNT in the presence of Hn33, and Hn33 alone (ρ<0.05). Statistical analysis using a one way ANOVA did not show significant differences between the binding of WGA, DrBoNT in the presence of Hn33, and Hn33 alone (ρ>0.05).

In order to understand the mechanism by which Hn33 facilitated DrBoNT-488 internalization, cell surface binding patterns of DrBoNT in the presence or absence of Hn33 were compared to that of Hn33 alone. HBE monolayers were treated with DrBoNT-488 (150 nM) in the absence or presence of unlabeled Hn33 (700 nM) and compared to Hn33-PacBlue (700 nM) alone. After 5 min cells were fixed, labeled and Z-stack images of the treated cells were obtained using confocal microscopy (Figs 4 and 5). Cell membranes were visualized with WGA-594 (red). The Z-stack images were compared to determine the location of the labeled proteins at the cell surface. Images of the cells treated with DrBoNT-488 in the absence of Hn33 showed only the labeled plasma membrane (red) (Fig 4a) with no DrBoNT-488 (green) (Fig 4b). This indicated that in the absence of Hn33, DrBoNT did not bind to the cell by 5 min whereas images of cells treated with DrBoNT-488 in the presence of Hn33 (Fig 4c) showed that DrBoNT bound across the cell surface by 5 min and the binding pattern was similar the binding of Hn33 alone (Fig 4d). The similar binding patterns of Hn33-PacBlue and DrBoNT-488 in the presence of unlabeled Hn33 suggested that Hn33 bound to the cell membrane with DrBoNT attaching to the bound Hn33, thereby allowing more DrBoNT to attach to cells [9, 10].

### Hn33 affected DrBoNT internalization into HBE cells

**Fig 6:**
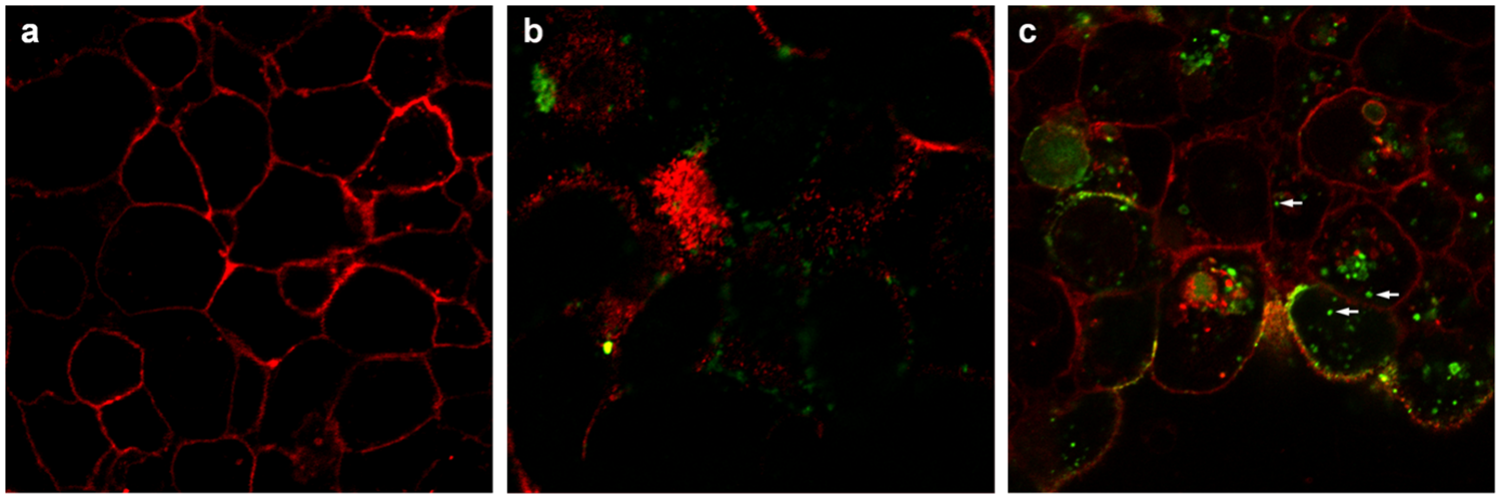
Effect of Hn33 on DrBoNT internalization. Polarized HBE cell monolayers were incubated with DrBoNT-488 (150 nM) in the absence or the presence of unlabeled Hn33 (700 nM) for 1.5 h and imaged using confocal microscope. Z-stacks of the cells were obtained using a 63X oil immersion objective with a 2X magnification. (a) shows the mid-section of control cells without the addition of any proteins; (b) shows the surface section of cells treated with DrBoNT-488 in the absence of Hn33; and (c) shows the mid-section of the cells treated with DrBoNT-488 in the presence of Hn33. DrBoNT-488 (green) is internalized in the presence of Hn33 via small vesicles as indicated by the white arrows (c), while it is localized to the surface of the cells in the absence of Hn33 (b). Cell membranes were labeled with WGA-594 (red).

**Fig 7:**
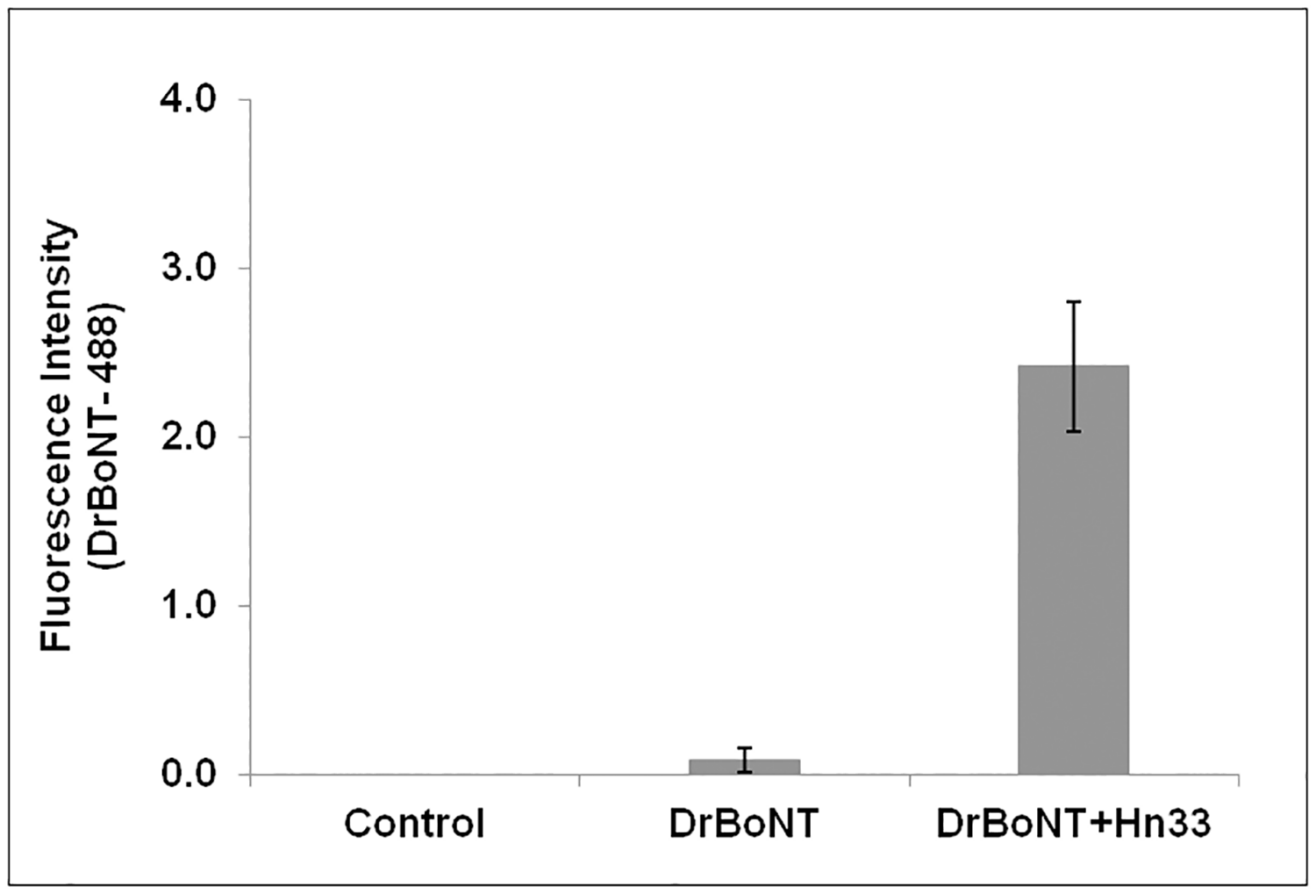
Comparison of DrBoNT internalization in the absence or presence of Hn33. The mean fluorescence intensities of cells for each treatment were measured and the background subtracted. The results were the average of five cells ± standard deviation. Significant difference (ρ<0.05) was observed between DrBoNT with Hn33 and DrBoNT-488 alone.

From the results shown in Fig 3, DrBoNT was detected in the basal media at 2 h but was not detected at 1 h (data not shown). Therefore, studies were performed to determine whether Hn33 increased DrBoNT internalization prior to detection in the basal media. Polarized HBE cells treated for 1.5 h, with DrBoNT-488 (150 nM) in the presence or absence unlabeled Hn33 (700 nM); cells were fixed and then assessed by confocal microscopy using the Z-stack analysis software. Mid-sections of the Z-stack were analyzed. HBE cells without any treatment were fixed and stained with WGA-594, and used as a control (Fig 6a). The surface section obtained from cells treated with DrBoNT-488 in the absence of Hn33 showed binding of a small DrBoNT-488 amount (green punctations) (Fig 6b). The mid-section of the Z-stack image showed the presence (green fluorescence) of very little DrBoNT (S1 Fig optical slices 5.14–8.99 μm), indicating a low amount of DrBoNT-488 internalization. Addition of Hn33 at a molar ratio of 1:4.6 (DrBoNT:Hn33) showed a dramatic increase in the signal on the surface sections as well as within the mid-sections of the image. Further analysis of the optical slices in the Z-stack suggested an enhancement of DrBoNT internalization in the presence of Hn33 (S2 Fig, section 3.00–4.71 μm). In the absence of Hn33, DrBoNT appeared to bind to the cell membrane but did not internalize for at least 1.5 h (Fig 6b). However, in the presence of Hn33, DrBoNT was found not only on the entire cell surface, but also was observed as small green vesicles inside the cells (Fig 6c), indicating that Hn33 facilitates the internalization of DrBoNT into HBE cells. The fluorescence intensities of DrBoNT-488 in the absence and presence of Hn33 was plotted (Fig 7) and statistical analysis using a Student’s t-test was performed. The results showed that the two were significantly different (over 10-fold; ρ<0.05).

**Fig 8:**
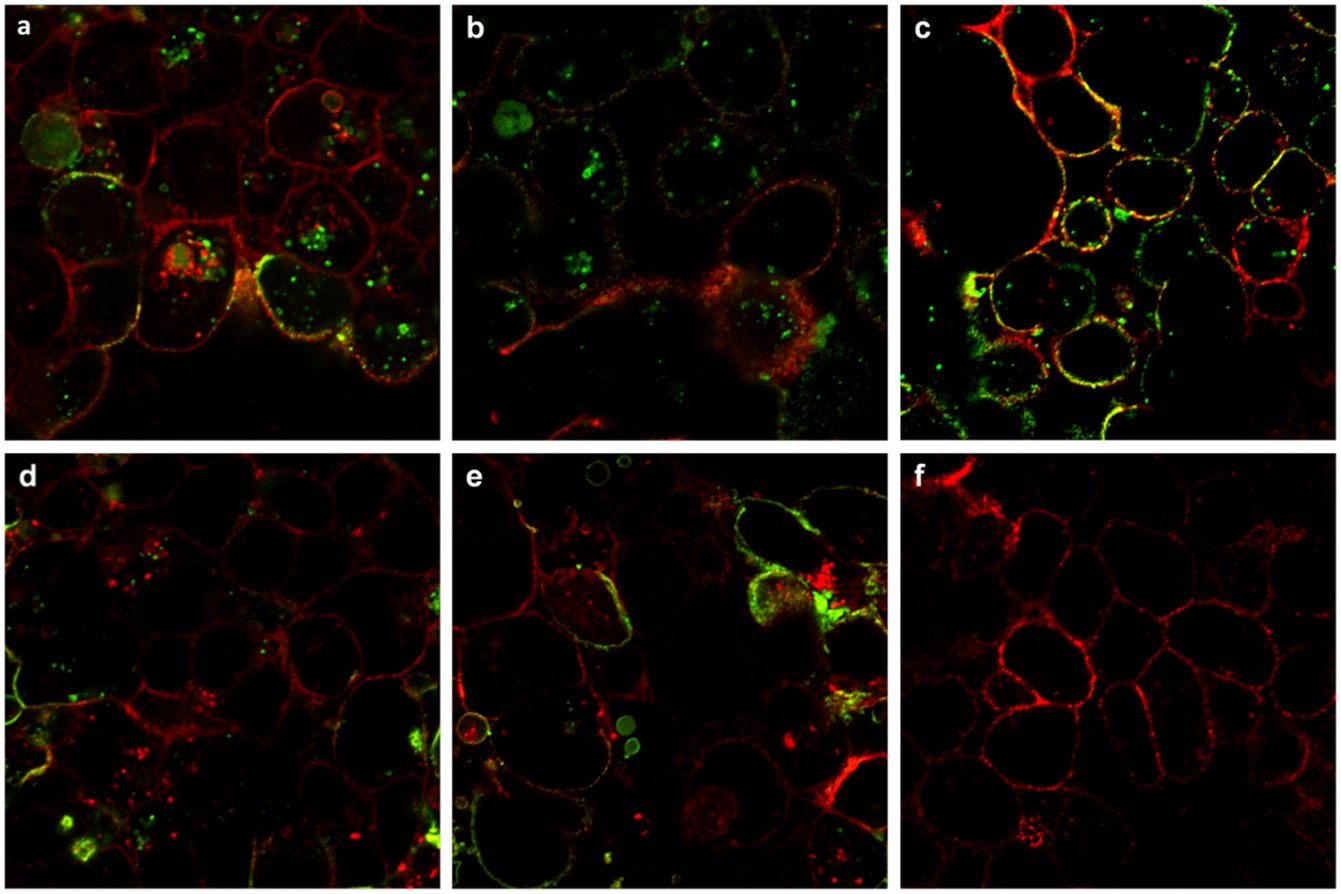
Internalization of DrBoNT in the presence of Hn33. (a-e): Polarized HBE cell monolayers were incubated with DrBoNT-488 (150 nM) in the presence of Hn33 (700 nM) for 1.5 h, 1 h, 30 min, 15 min and 5 min, respectively; (f): Control cells without DrBoNT or Hn33. Cell membrane was visualized with WGA-594 (red) and images were obtained using a 63X oil immersion objective with a 2X magnification.

**Fig 9:**
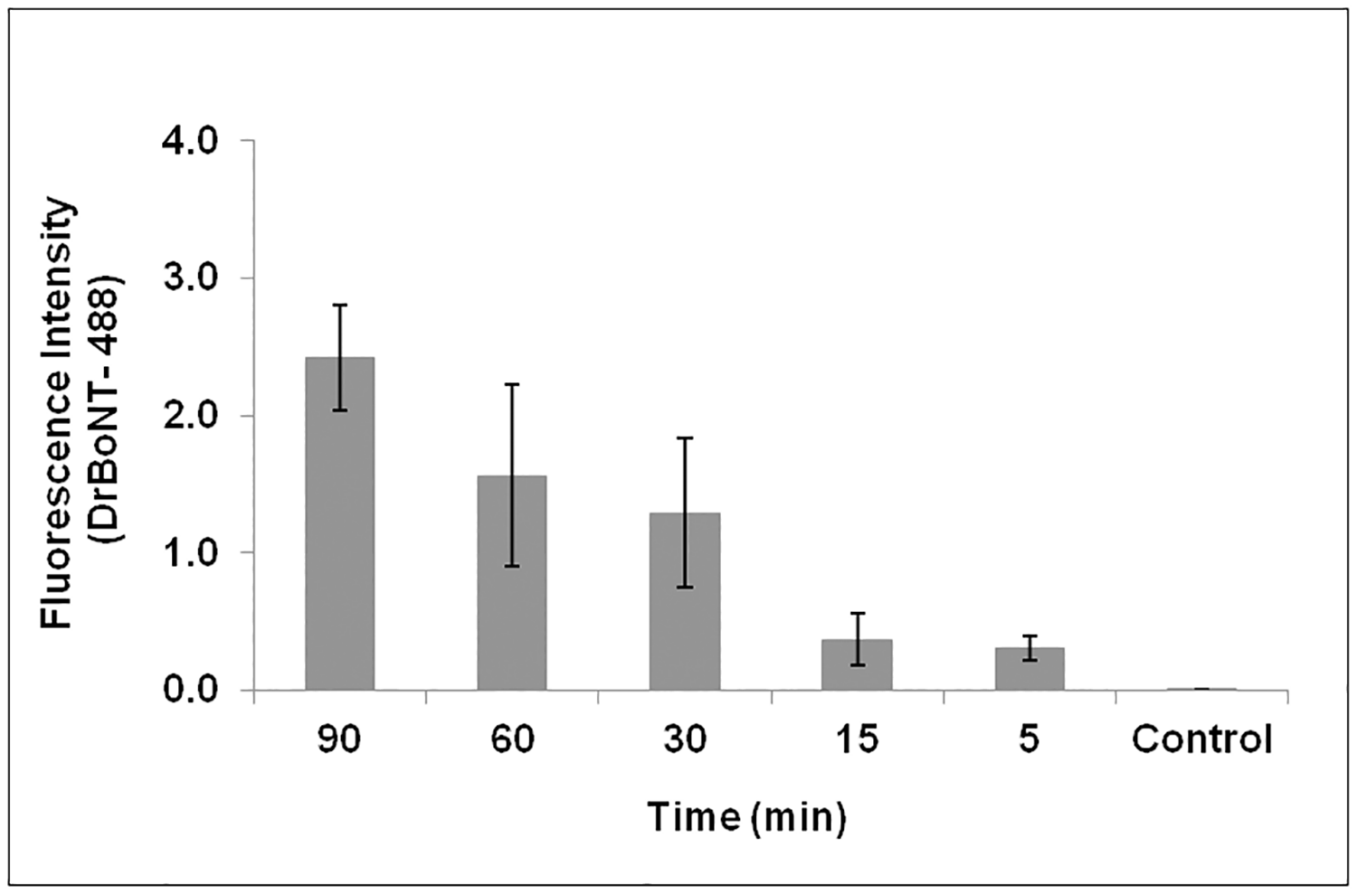
Comparison of DrBoNT internalization in the presence of Hn33 at various time points. The mean fluorescence intensities of cells for each treatment were measured and the background subtracted. The results were the average of five cells ± standard deviation. Statistical analysis performed using a one-way ANOVA showed that the data was statistically significant (ρ<0.05).

From Figs 6 and 7, Hn33 was shown to affect DrBoNT internalization and appeared to provide an additional route for DrBoNT transcytosis. For further examination of internalization, cells were incubated with DrBoNT-488 and Hn33 for various time points between 1.5 h and 5 min. At each time point cells were fixed and examined by confocal microscopy. From the Z-stack, the mid-sections were analyzed. Internalization was observed in the cells as small green punctations at all time points, including at 5 min (Fig 8a–8e). Cells without any treatment were used as a control (Fig 8f). In the absence of Hn33, the earliest time point at which DrBoNT could be was observed in vesicles was 1.5 h (S1 Fig) but in the presence of Hn33, DrBoNT internalization began as early as 5 min (Fig 8e) and increased in a time-dependent manner as seen in the images in Fig 8a, 8b, 8c and 8d. The fluorescence intensities of DrBoNT-488 within cells was plotted over time (Fig 9) and statistical analysis using a one way ANOVA showed the significant differences between the time points (ρ<0.05).

### DrBoNT and Hn33 colocalized in vesicles

**Fig 10:**
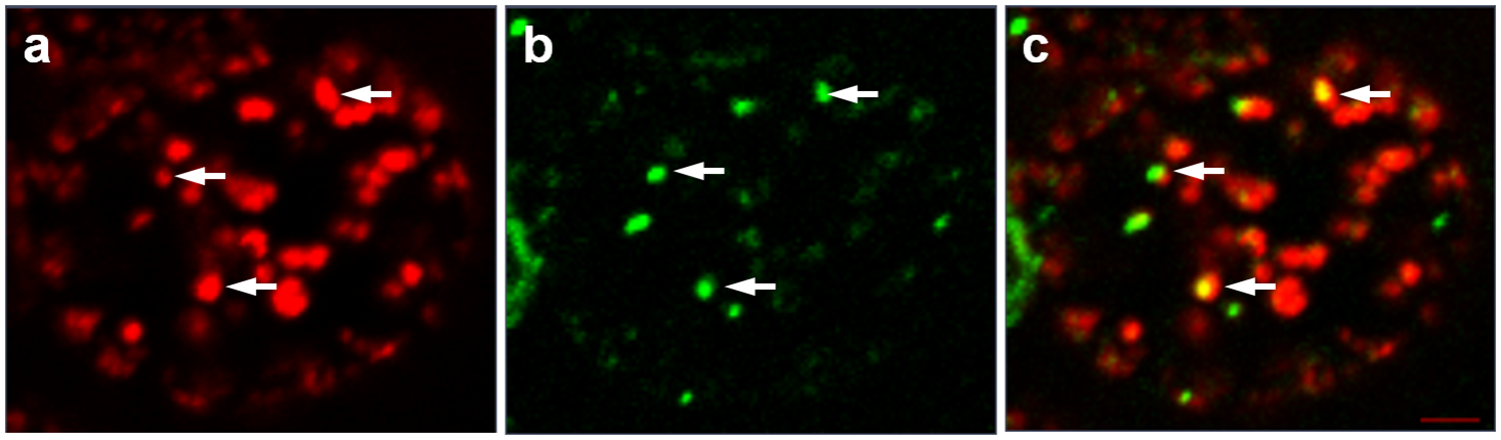
Labeled DrBoNT internalization in the presence of labeled Hn33. Polarized HBE cell monolayers were incubated with DrBoNT-488 and Hn33-PacBlue for 1.5 h. Cells were fixed and then imaged. (a) Hn33-PacBlue (red); (b) DrBoNT-488 (green), and (c) Hn33-PacBlue and DrBoNT-488 merged. Arrows point to the punctations showing colocalization. (Manders coefficient = 0.6) Red bar = 2 μM.

Hn33 was not labeled in previous experiments, and therefore, in order to trace the path of Hn33 as well as DrBoNT, experiments were conducted using Hn33-PacBlue that was incubated with DrBoNT-488 for 1.5 h. The images were examined in their respective channels (Fig 10a and 10b) and as a merged image (Fig 10c). For analysis, Hn33-PacBlue was pseudo-colored red for effective visualization of protein colocalization. Hn33 internalization was observed as small red punctations in distinct vesicles within the cell (Fig 10a) while DrBoNT was observed as green punctations that appeared to localize in the same vesicles as Hn33 (Fig 10b). When the two channels were viewed as a merged image, DrBoNT was shown to colocalize with Hn33 (yellow punctations within the vesicles (Fig 10c). Colocalization analysis yielded a Manders coefficient of 0.6. These analyses indicated that DrBoNT bound to Hn33 and then moved across the cell together.

### BoNT/A complex internalized more readily than BoNT/A in HT-29 intestinal epithelial cells

To determine the role of NAPs in the trafficking of BoNT/A across epithelial barriers, 300 nM BoNT/A and BoNT/A complex, individually labeled with AlexaFluor 488 (green), were incubated (37°C) with intestinal epithelial (HT-29) cells for different time points (30 min, 3 h, 6 h) and examined for binding and internalization of the labeled proteins using confocal laser scanning microscopy. Specific labeling of the plasma membrane was carried out with WGA-594 (red) to demarcate the cell boundaries so that the binding of AlexaFluor 488-labeled proteins to the cell membrane can be distinguished from their internalization within the cell compartment.

**Fig 11:**
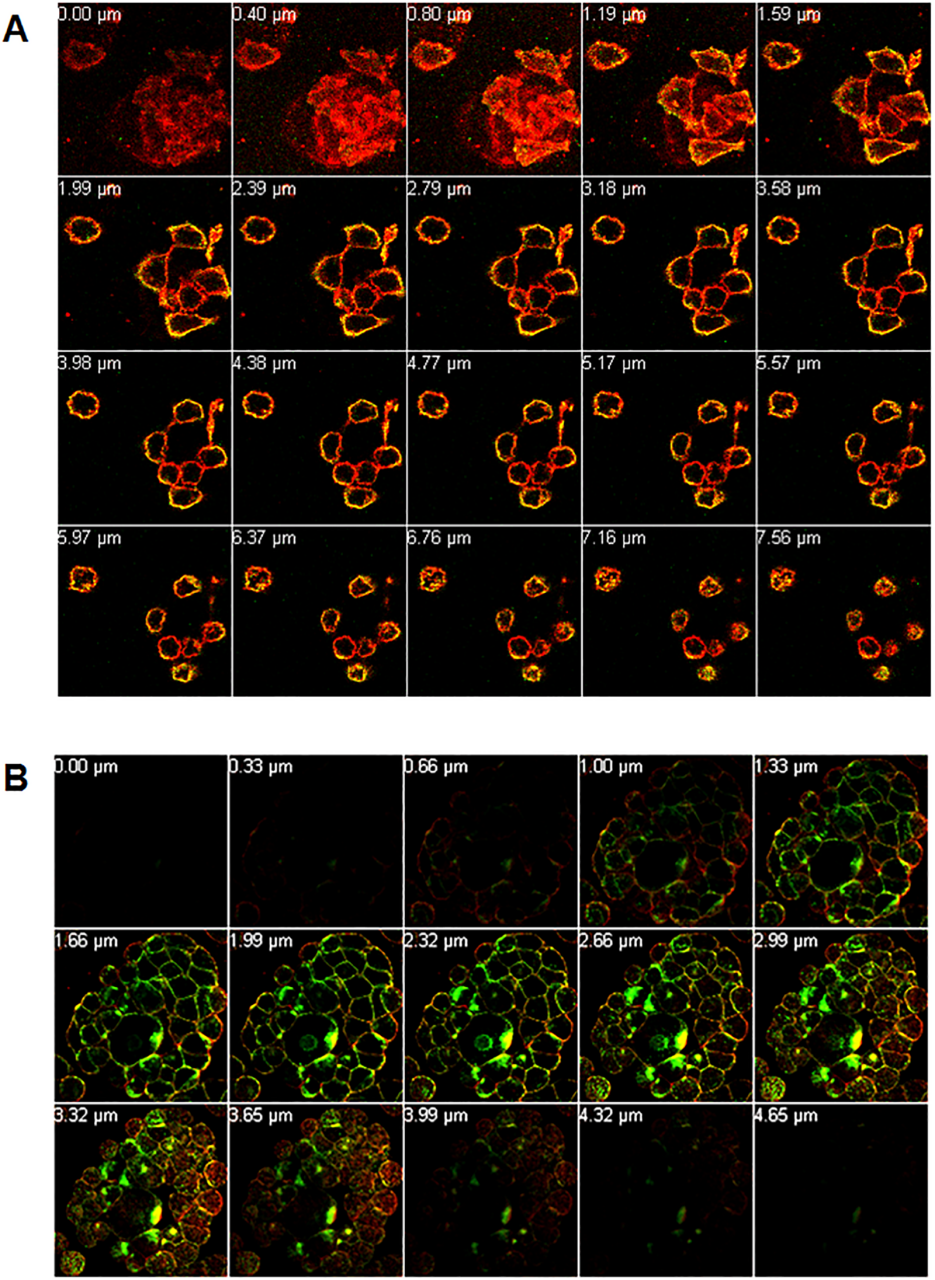
BoNT/A complex internalized faster than BoNT/A in HT-29 intestinal epithelial cells. Cells grown on coverslips were incubated with 300 nM BoNT/A-488 (A) or BoNT/A complex-488 (B) for 30 min, washed with HBSS and labeled plasma membrane with WGA-594 (red), washed, fixed and mounted for confocal microscopy as described in methods. Images obtained using a 63X oil immersion objective. Z-stack images showed BoNT/A only on the cell surface at 30 min, while at the same time, BoNT/A complex showed internalization into the cell compartment. Optical slices were viewed from basal to apical side of the cells.

Optical sectioning using 63X magnification of intestinal epithelial cells incubated with BoNT/A-488 or BoNT/A complex-488 for various time points was carried out by performing Z-stack analysis on the sample to distinguish between binding of the labeled protein to the cell surface and internalization into the cells. Although both BoNT/A and BoNT/A complex were internalized when incubated for 3 or 6 h, more complex was internalized than the toxin alone (data not shown). Z-stack images of HT-29 cells incubated with BoNT/A-488 (Fig 11A) were examined by viewing optical section of the cells, going from the bottom surface of the cells that are attached to the cover glass to the top surface of the cells that is in contact with the mounting medium. Each optical slice of 0.4 μm thickness from the Z-stack shows a digitally constructed image of the cross-section of the cells in that particular Z-plane. Cells showed strong green signal from the AlexaFluor 488 co-localized with the red signal from the AlexaFluor 594-WGA membrane dye, resulting in yellow signal in all the optical slices of the Z-stack. No green signal was observed in the interior of the cell at this time point. This indicates that BoNT/A-488 was localized only to the cell surface without any internalization of the protein into the cell. Thus, at 30 min, BoNT/A exhibited high binding but showed no internalization into the cells. However, at the same time point, HT-29 cells incubated with BoNT/A complex-488 showed strong green fluorescence localized with the red fluorescence from the membrane dye. BoNT/A complexes were also observed inside the cell as shown in the mid slices of the Z-stack (Fig 11B). These observations indicate that by 30 min, BoNT/A complex showed not only binding to the surface of the epithelial cells, but also significant internalization, suggesting that the presence of NAPs allows the internalization more readily.

### DrBoNT bound directly to Hn33

**Fig 12:**
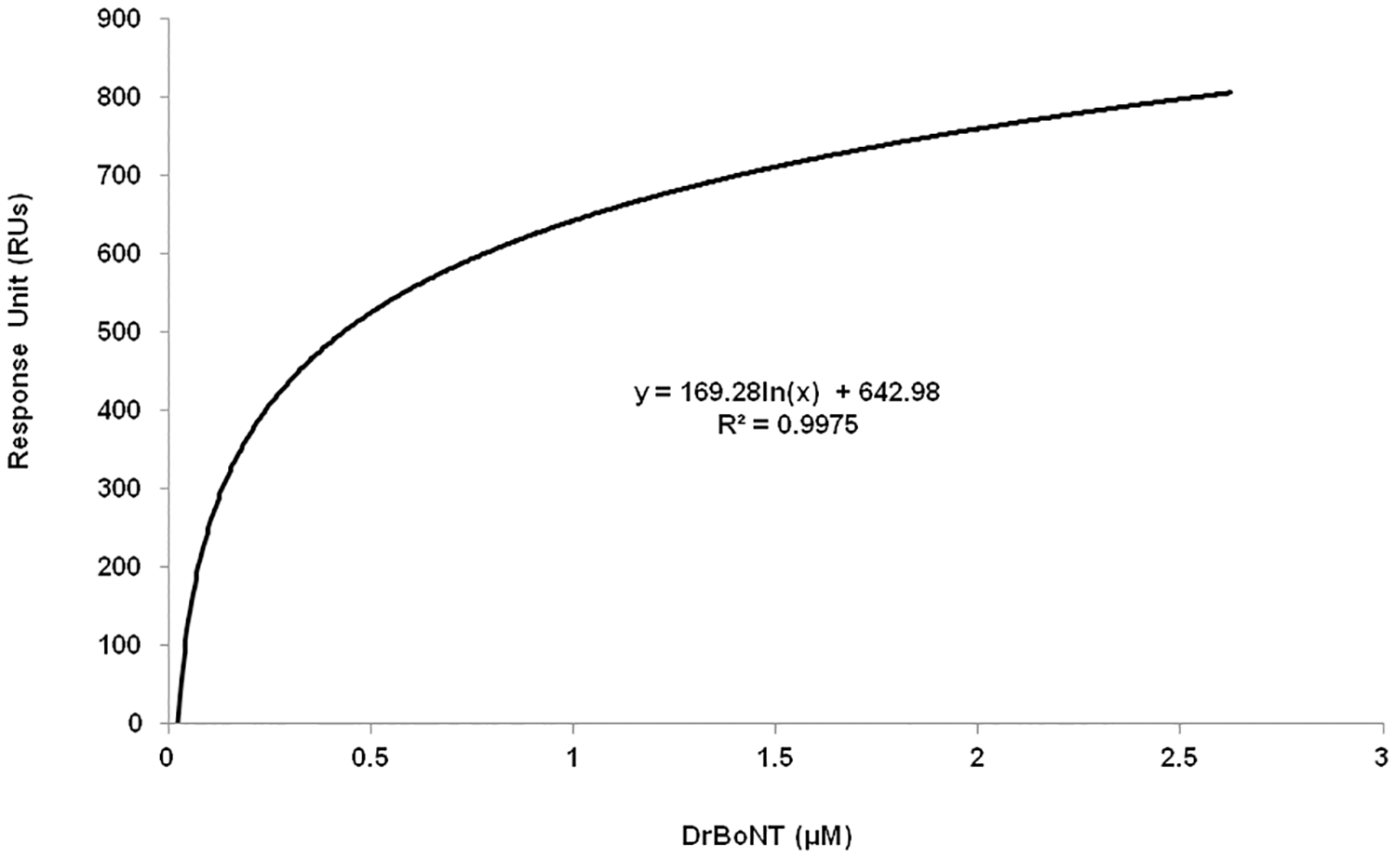
Binding analysis of DrBoNT and Hn33 using surface plasmon resonance. The DrBoNT and Hn33 binding rate constant was determined using a Biacore T100 SPR. The SPR-derived steady-state binding of DrBoNT to Hn33 protein immobilized by amine coupling to a carboxymethylated dextran-coated CM3 sensor chip.

The binding affinity of Hn33 and DrBoNT was measured using surface plasmon resonance (SPR) using a Biacore 100T instrument. As determined with isothermal calorimetry (ITC) [28], the dissociation constant (K_D_) for Hn33-BoNT/A toxin binding has been reported as 0.4 μM. The K_D_ was determined in this study by immobilizing ligand Hn33 on the CM_3_ chip through amine coupling to different concentrations of the analyte DrBoNT. The binding affinity, K_D_ = 0.13 μM ± 0.03, using the binding steady state kinetic model (Fig 12) in SPR measurements, was similar to the previously reported value of 0.4 μM [40]. These studies showed that the binding affinity of Hn33 to BoNT/A toxin was similar to the binding affinity of Hn33 to DrBoNT (a mutated toxin). Because the k_on_ and k_off_ rates of kinetic binding were extremely rapid, the binding analysis was based on equilibrium steady-state affinity measurements, using maximal response values during the addition of DrBoNT. The stoichiometric ratio of DrBoNT to Hn33 was 1 to 2.3 using the Biacore data analysis Eq (1).

Rligand=RmaxSmxMWligandMWanalyte (1)

where, R_ligand_—amount of ligand to be immobilized, R_max_—maximum response, S_m_—stoichiometric ratio (number of binding sites per ligand), valency of ligand, MW_ligand_ and MW_analyte_—molecular weights of ligand and analyte respectively.

## Discussion

As part of the neurotoxin-associated proteins (NAPs), hemagglutinin 33 (Hn33) forms progenitor toxin complexes (PTCs) that protect the toxin from harsh environments as evidenced by the fact that, when given orally, PTCs are 10- to-100 fold more toxic than free BoNTs [16, 17]. While the larger molecular weight (120 kDa) nontoxicnonhemagglutinin protein (NTNH) has been shown to form a tight protective complex with BoNT/A, the smaller 33 kDa Hn33 has also been shown to stabilize and protect the toxin from proteolysis [21, 41, 42]. The barrel-shaped Hn33 protein is composed of 2 β trefoil domains linked by an α-helix. The β trefoil domain is similar in structure to those in other proteins such as lectins that bind to oligosaccharides found in the membranes of many cells [39, 43]. Hn33 binds to oligosaccharides in mammalian erythrocytes and studies show that, like many lectins, the hemagglutinin binds nonspecifically to cell membrane oligosaccharides [30]. Our studies show that in addition to membrane binding, Hn33 binds to BoNT/A at a high affinity (Fig 12).

In addition to their ability to protect the toxin, NAPs, particularly the hemagglutinins (Hns), have also been shown to cross epithelial cells and possibly affect the integrity of intestinal epithelial cell barriers by binding to proteins on the basal side of the membrane [44] Hn binding to the basal side, which occurs 48 h after intoxication, appears to disrupt the epithelial cell barrier allowing more toxin to move across the intestines via a paracellular pathway. Our results, however, show that during the first 24 h, the toxin crosses the bronchial epithelial cell barrier by transcytosis and that Hn33 enhances the passage of DrBoNT through HBE cells without disrupting the epithelial barrier (Figs 1 and 3).

In order to monitor transcytosis during the first 8 h, a highly sensitive, non-radioactive assay in which toxin, labeled with a dye that fluoresces in the near infrared range, was used to follow DrBoNT/A transcytosis 2–8 h post-addition of the labeled proteins to the apical side. The integrity of tight junctions was not compromised by DrBoNT, and Hn33 treatment of HBE cell monolayers during the first 24 h did not affect tight junction integrity as visualized by the anti-ZO-1 IgG (Fig 1). In addition, other measurements of membrane integrity, such as a high electrical resistance (TEER) and no leakage of DEX-680, further indicates that the proteins did not cross the cell monolayers via a paracellular route but moved across the cell by transcytosis.

When HBE cell monolayers were treated with DrBoNT in the presence of varying concentrations of Hn33 (Fig 3), the amount of DrBoNT detected in the basal medium increased proportionally as the amount of Hn33 increased. Examination of cell monolayers by confocal microscopy showed that within 5 min, in the presence of Hn33, the entire cell surface was coated with DrBoNT (Fig 4c). Internalization was seen as vesicles inside the cells in a pattern similar to that seen with Hn33-PacBlue (Fig 10). The same enhancement of transcytosis was not observed with SEB, another protein of similar molecular weight (Fig 3), indicating the unique ability of Hn33 to bind and associate with DrBoNT and thereby facilitate DrBoNT entry into cells.

BoNT/A has been reported to undergo transcytosis from apical to basolateral surface of T84 intestinal cell monolayers in a clathrin-dependent manner with a migration pathway close to the cell periphery [37, 45]. Previous studies by Ahsan and colleagues [35] have demonstrated transcytosis of toxin labeled with AlexaFluor 488 through T-84 cell monolayers within 5 to 20 min at 37°C; however, the transcytosis was observed only after the toxin-dye complex had been preincubated with the cells for 90 min at 3°C [35]. Other investigations show that BoNT/A binds dually to gangliosides and SV2 as a means of initiating the transcytosis process across human intestinal epithelial cells, similar to investigations showing that BoNT binds tightly to neuronal cells via two receptors [14, 46, 47]. SV2 was not detected on the HBE cell surface either by western blot or confocal imaging, and pretreatment of the cell monolayers with anti-SV2-IgG did not affect BoNT transcytosis indicating that SV2 is not involved in BoNT transcytosis across HBE cells (data not shown). Our studies (Fig 6b) show that BoNT binding on the cell surface occurred in localized regions which would indicate that there are relatively few BoNT receptors on HBE cells. Lacking an SV2 receptor, the presence of Hn33 on the cell surface may help stabilize the toxin and thereby facilitate BoNT transcytosis. Although Hn33 presence may help anchor BoNT to the cell surface, the binding and internalization patterns of DrBoNT alone appeared different from that of DrBoNT in the presence of Hn33 (Figs 4–7). After a 1.5 h (37°C) incubation, only slight DrBoNT internalization was observed (Fig 6, S1 Fig). Internalization of DrBoNT occurs close to the periphery of the cell membrane around the nucleus, similar to results reported in an earlier study [25]. When DrBoNT and Hn33 were incubated for 5 min, the cells appeared to be coated with DrBoNT (Fig 4c). Additionally, distinct internalization could be observed as punctate vesicles in the cells at 5 min (Fig 8e) after addition of the proteins. In the presence of Hn33, DrBoNT did not localize in discrete regions on the cell surface, but appeared to coat the cell surface, with internalization distributed throughout the cytoplasmic region of the cell (S2 Fig).

Studies performed (Figs 4 and 10), in which Hn33 was labeled with Pacific Blue showed binding and internalization patterns similar to that of DrBoNT in the presence of Hn33, suggesting that Hn33 bound nonspecifically to the cell surface while DrBoNT associated with the bound Hn33 and was then internalized with Hn33. Previous studies show that Hn33 binds to BoNT/A [40] and the present data suggest that Hn33 binding to HBE cell membranes may not only help anchor BoNT to the cell, but also provide more binding sites, thus dramatically increasing DrBoNT binding to HBE cells. This binding process may be responsible for reducing the time for internalization and subsequent transcytosis. A similar pattern of binding and internalization was also observed when reconstituted DrBoNT-488-NAPs complex was incubated with HBE cells (data not shown), supporting the premise that Hn33 within NAPs moiety plays a role in the binding and internalization of the DrBoNT-NAPs complex. Similarly, we have demonstrated that BoNT/A complex internalized faster than BoNT/A in HT-29 cells in which there is a significant increase in BoNT/A complex internalization (Fig 11B) when compared to BoNT/A internalization at the same time point (Fig 11A), indicating that an additional role of NAPs may be to enhance BoNT/A internalization into HT-29 cells. These results are similar to a recent study by Lam et al. [48], using CaCo2 cells and mouse intestinal tissues, who show that the presence of NAPs increases the rate of toxin internalization. Lam et al. [38], however, focused more on the role of BoNT/A NTNH and HA-70 components. Previous studies have shown that in case of BoNT/C, NAPs are essential for binding and absorption of the toxin into the intestinal epithelial cells [15, 49] and, in case of BoNT/A and BoNT/B, NAPs play a role in disrupting the paracellular barrier when incubated with the cells for longer times [18]. Our confocal images (Fig 11A and 11B) showed that the BoNT/A complex was internalized more rapidly into the cells when compared to BoNT/A alone. The recent work by Lam et al. [48], which used much lower toxin concentrations than used in our study, show similar conclusions, except our data establishes differences between BoNT/A and BoNT/A complex within minutes whereas Lam et al. [48] data show these differences occur in a few hours. These studies together suggest that internalization of BoNT/A complex is similar in many epithelial cell models (HBE, HT-29, CaCo2 or intestinal villi). The role of Hn-33 examined in the present study was unique for the following reasons: (1) Treatment with higher concentration of Hn-33 (666.7 nM) did not alter the cell-cell tight junctions, similar to the conditions of Lam et al. [48] but in contrast to the observations of Matsumura et al. [44] and Jin et al. [18]. This allowed us to examine the role of Hn-33 in the transcytosis process rather than by paracellular transport. (2) The extent of transcytosis facilitation was quite dramatic when compared to the observations of Lam et al. [48] and may be a reflection of concentrations used, specific effects of the Hn-33, and the epithelial cells used. Both NTNH and HA-70 had only marginal effects on BoNT/A internalization [48], whereas the Hn-33 data in this study shows dramatic effects. (3) Finally, Hn-33 influences the internalization and transcytosis of BoNT/A, even when the recombinant Hn33 was incubated with DrBoNT, and not as a component of the BoNT/A complex. Receptor-mediated BoNT/A transcytosis in intestinal cell monolayers that is not enhanced by NAPs have also been reported [14]. Our results show that, alone, DrBoNT undergoes receptor-mediated transcytosis in HBE cells (Table 2). In the presence of Hn33, however, DrBoNT follows an additional path as observed in the confocal data showing DrBoNT internalization in the presence and absence of Hn33 (Figs 6–10). Couesnon and co-workers [14] show that Hn33 had little effect on BoNT/A transcytosis across intestinal epithelial cells, however their studies utilized the NAP complex rather than Hn33 alone.

Although Hn33 does not bind to a specific membrane receptor, Hn33 binds to various oligosaccharides on the cell surface, and, similar to other agglutinins initiates transcytosis via an adsorptive process (Fig 4) [38, 43]. Wheat germ agglutinin shows a similar binding to the cell surface (Fig 4a) and is reported to undergo adsorptive transcytosis [39, 50]. While Hn33 binding and transcytosis is similar to other lectins, BoNT/A binding to Hn33 adsorbed on the cell surface has not been reported and provides BoNT with an additional, more rapid pathway for moving across an epithelial cell. Structural studies using negative stain electron microscopy have shown that in case of BoNT/A1 and BoNT/B, PTCs are flexible, three armed structures, wherein the toxin does not directly interact with the Hns but via the NTNH, suggesting that Hns may not only be required for toxin protection, but may also be important for transport into cells through cell surface interactions [51]. Our data and the work of Benefield and colleagues [51] show that Hns are important for facilitating transcytosis. While our data is in contrast to the structural model proposed for the entire BoNT/A PTC complex [41, 52, 53], direct binding of Hn33 to the toxin has been observed and occurs in the absence of other NAPs [40]. Direct binding was further confirmed in this study with SPR analysis of the binding between DrBoNT and Hn-33, showing a strong affinity, with a dissociation constant of 130 nM (Fig 12). Hn-33 binding to BoNT/A needs to be further examined with techniques such as x-ray crystallography in order to understand the molecular basis of such interactions.

The prominent role of Hn33 in the translocation across the epithelial barriers in intestinal and bronchial tissues underscores its potential to accelerate the toxin uptake when used as a biothreat agent, and thus provides a critical target for developing countermeasures, such as developing small molecule inhibitors against Hn33 to prevent intentional BoNTs exposure to a large group of individuals. Further studies need to be conducted to determine the binding domains of Hn33 to DrBoNT, and to determine the exact mechanism by which Hn33 interacts with the cell membrane. Information from these studies can also be used to exploit Hn33 as a drug delivery vehicle, which, in conjunction with DrBoNT, can be used for targeted neuronal drug delivery via inhalational route.

## Supporting information

S1 Fig Z-Stack of HBE cells incubated with DrBoNT-488 for 1.5 h. Cells were imaged using Zeiss 710 confocal laser scanning microscope. Z-stacks of the cells were obtained using a 63X oil immersion objective with a 2X magnification. Each optical slice is 0.43 μm and the distance from the top surface of the cells along the Z-plane is denoted on the left hand corner of each optical slice. DrBoNT-488 (green) is localized to the surface of the cells in the absence of Hn33. Cell membrane is labeled with WGA-AlexaFluor 594 (red).
(TIF)

S2 Fig Z-Stack of HBE cells incubated with DrBoNT-488 in the presence of Hn33 for 1.5h. Cells were imaged using Zeiss 710 confocal laser scanning microscope. Z-stacks of the cells were obtained using a 63X oil immersion objective with a 2X magnification. Each optical slice is 0.43 μm and the distance from the top surface of the cells along the Z-plane is denoted on the left hand corner of each optical slice. Optical slices 0.00–0.86 μm denotes the apical surface of the cells and 3.00–4.71 μm represents the mid-section of the cells. DrBoNT-488 (green) is seen bound to the surface of the cells, as well as internalized in the presence of Hn33 via small vesicles. Cell membrane is labeled with WGA-AlexaFluor 594 (red).
(TIF)
