# Supplementary figures and images for: Role of critical elements in botulinum neurotoxin complex in toxin routing across intestinal and bronchial barriers
Source: PLoS One. 2018 Jul 5;13(7):e0199524. doi: 10.1371/journal.pone.0199524 (PMC6033393; doi:10.1371/journal.pone.0199524)

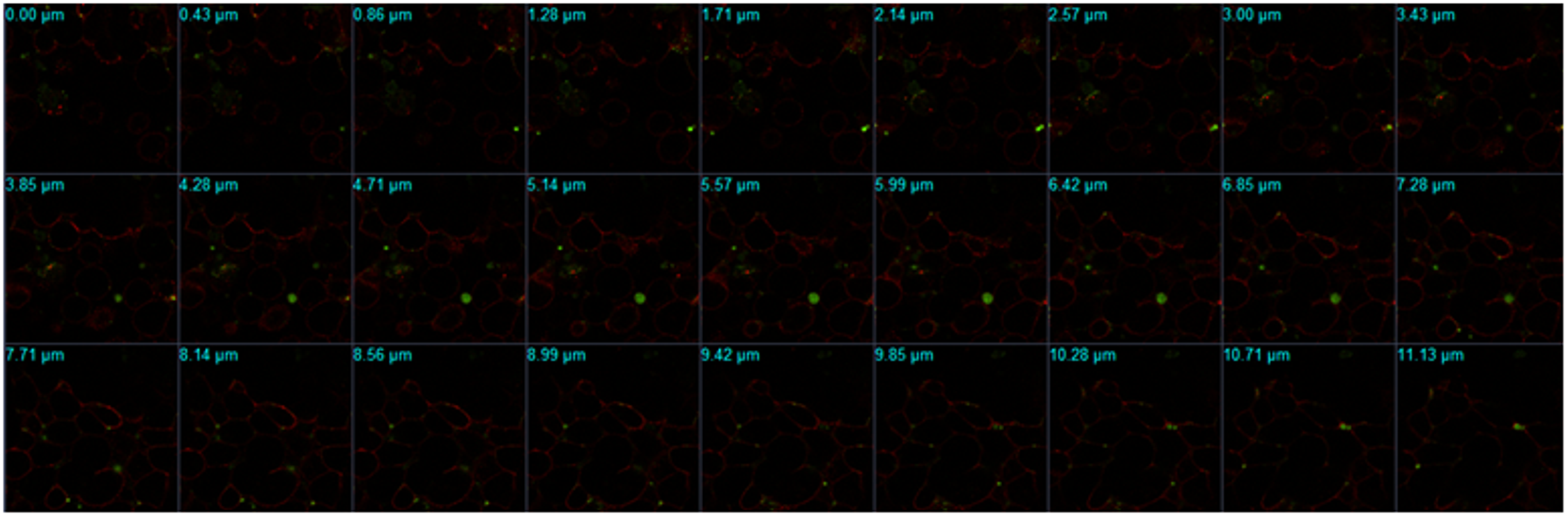

Supplement: S1 Fig — Cells were imaged using Zeiss 710 confocal laser scanning microscope. Z-stacks of the cells were obtained using a 63X oil immersion objective with a 2X magnification. Each optical slice is 0.43 μm and the distance from the top surface of the cells along the Z-plane is denoted on the left hand corner of each optical slice. DrBoNT-488 (green) is localized to the surface of the cells in the absence of Hn33. Cell membrane is labeled with WGA-AlexaFluor 594 (red). (TIF) [file pone.0199524.s001.tif]

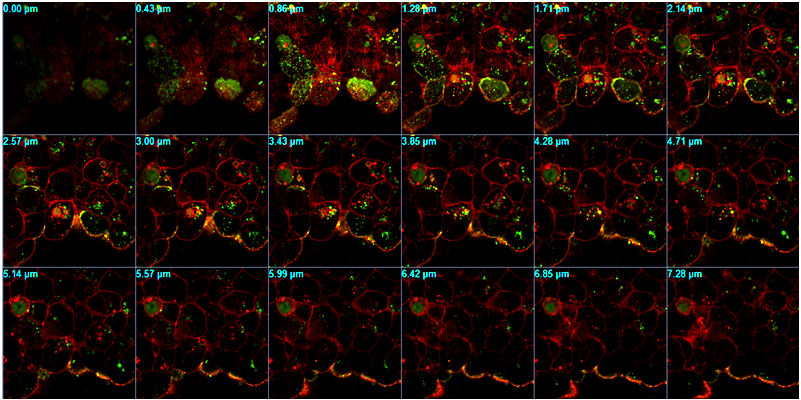

Supplement: S2 Fig — Cells were imaged using Zeiss 710 confocal laser scanning microscope. Z-stacks of the cells were obtained using a 63X oil immersion objective with a 2X magnification. Each optical slice is 0.43 μm and the distance from the top surface of the cells along the Z-plane is denoted on the left hand corner of each optical slice. Optical slices 0.00–0.86 μm denotes the apical surface of the cells and 3.00–4.71 μm represents the mid-section of the cells. DrBoNT-488 (green) is seen bound to the surface of the cells, as well as internalized in the presence of Hn33 via small vesicles. Cell membrane is labeled with WGA-AlexaFluor 594 (red). (TIF) [file pone.0199524.s002.tif]
